# Reversible Advanced Liver Fibrosis in Pediatric Constrictive Pericarditis

**DOI:** 10.1016/j.jaccas.2026.109911

**Published:** 2026-08-20

**Authors:** Svetlana A. Konchina, Dmitry V. Kovalev, Lyale Ch. Yanybaeva, Victor B. Samsonov, Nikolay S. Chikin, Timur Y. Danilov

**Affiliations:** Congenital Heart Diseases Department, Federal State Budgetary Institution “National Medical Research Center of Cardiovascular Surgery Named After A.N. Bakulev”, Ministry of Health of the Russian Federation, Moscow, Russia

**Keywords:** chronic constrictive pericarditis, congestive hepatopathy, pediatric pericarditis, pericardiectomy, pericarditis

## Abstract

**Background:**

Chronic constrictive pericarditis is a rare cause of heart failure in children and may present with nonspecific symptoms. Chronic venous congestion can result in liver injury mimicking cirrhosis.

**Case Summary:**

An 8-year-old boy presented with exercise intolerance, dyspnea, and longstanding gastrointestinal symptoms leading to a diagnostic delay of nearly 5 years. Evaluation revealed hepatomegaly and advanced liver fibrosis (METAVIR F4) on elastography, despite preserved liver function. Multimodality cardiac imaging demonstrated features consistent with constrictive pericarditis. The patient underwent successful pericardiectomy. At 3-month follow-up, liver stiffness regressed to METAVIR F1, indicating reversal of congestion-related fibrosis.

**Discussion:**

Diagnosis of pediatric constrictive pericarditis remains challenging. This case demonstrates that advanced fibrosis due to venous congestion may be reversible after correction of the underlying hemodynamic abnormality.

**Take-Home Messages:**

Constrictive pericarditis should be considered in children with systemic venous congestion. Timely surgical treatment may lead to reversal of liver fibrosis.

## Visual Summary

**Figure:**
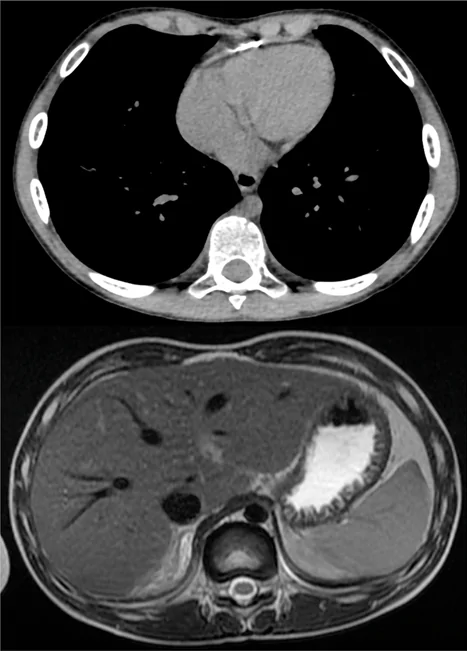

## History of Presentation

An 8-year-old boy presented to the cardiac surgery department with complaints of dyspnea, exercise intolerance, perioral cyanosis during physical activity, and recurrent episodes of abdominal pain with nausea. On physical examination he was afebrile with normal heart rate and blood pressure for age. No pedal edema or ascites were noticed. Cardiopulmonary auscultation revealed normal heart sounds with no murmurs or friction rub. However, jugular venous distention and marked hepatomegaly (+3 cm below the right costal margin) were suggestive of systemic venous congestion and right-sided heart failure.

## Past Medical History

The patient had been symptomatic since the age of 2.5 years, initially presenting with vomiting and significant fatigue. Multiple attempts for treatment of foodborne illness, gastroduodenitis, gastroesophageal reflux disease, and helminthiasis did not show clinical improvement, leading to a prolonged diagnostic delay of nearly 5 years. Initial transthoracic echocardiography (TTE) performed at an outside institution, without dedicated respiratory assessment, was reported as normal.

At 8 years of age, abdominal ultrasound followed by magnetic resonance imaging revealed hepatomegaly with periportal fibrosis and edema (Figure 1), with no splenomegaly (vertical length of 8 cm). Transient elastography performed at that time demonstrated a median liver stiffness of 40 kPa, consistent with METAVIR stage F4 cirrhosis.

**Figure 1:**
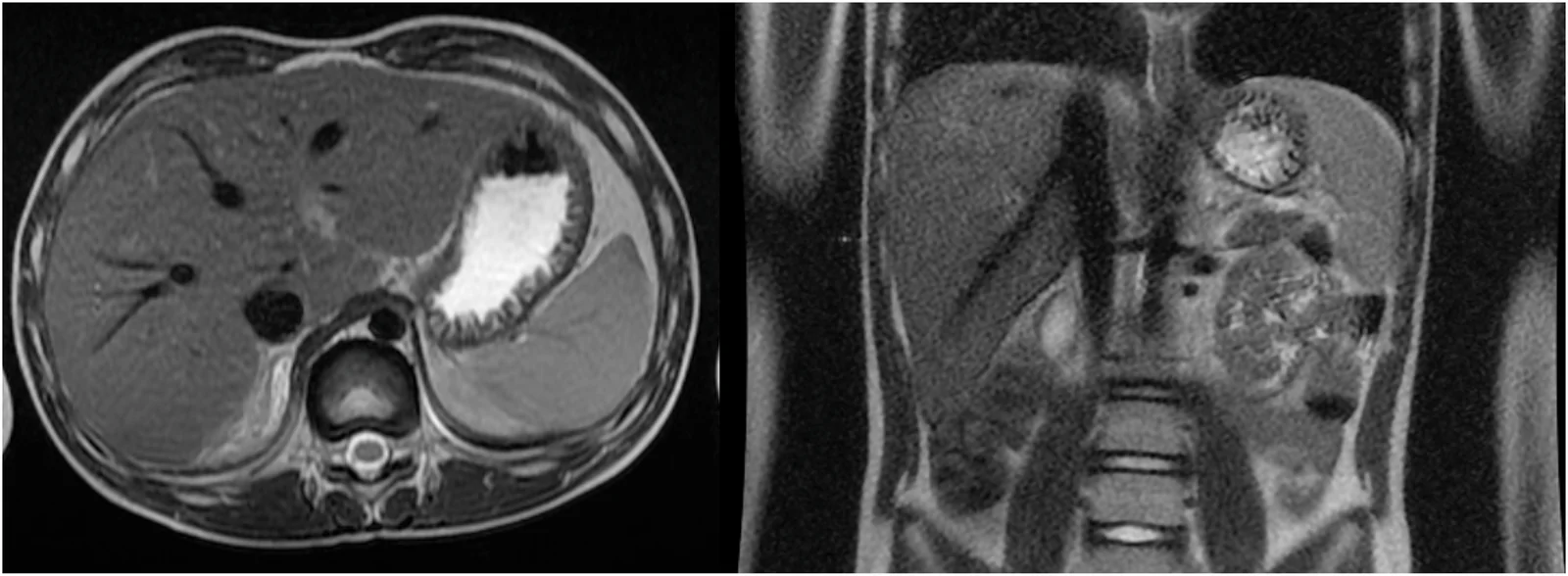
Abdominal Magnetic Resonance Imaging at Initial Evaluation Magnetic resonance imaging demonstrates hepatomegaly (vertical dimension of the right lobe 145 mm) with diffusely heterogenous parenchyma and perivascular fibrosis. Portal vein diameter is 6 mm; hepatic veins are dilated to 11 mm.

## Differential Diagnosis

Infectious hepatitis, autoimmune liver disease, and infiltrative disorders were ruled out with extensive laboratory workup. Liver biochemistry revealed normal transaminases (alanine aminotransferase 20 U/L, aspartate aminotransferase 34 U/L), mild coagulation abnormalities, including prolonged prothrombin time (20 s; reference 14-15.4 s) and reduced prothrombin index (54%; reference: 70%-120%), with preserved albumin (46 g/L) and normal platelet count (310 × 10^9^/L). This pattern was inconsistent with primary cirrhosis of this severity. The combination of advanced fibrosis on elastography, absence of synthetic liver dysfunction, and clinical signs of systemic venous congestion raised suspicion for a cardiac etiology. Both restrictive cardiomyopathy and constrictive pericarditis were included in the differential diagnosis, prompting dedicated cardiovascular evaluation.

## Investigations

Additional laboratory testing revealed an elevated N-terminal pro–B-type natriuretic peptide level (184 pg/mL, above the upper limit of normal for age), consistent with increased right-sided filling pressures. However, the level was only moderately elevated and lower than typically observed in primary myocardial disease, such as restrictive cardiomyopathy, supporting a constrictive rather than myopathic etiology.^1^

TTE with dedicated assessment for constriction demonstrated classic features of constrictive pathophysiology, including respirophasic variation in mitral and tricuspid inflow exceeding 30%, increased medial mitral annular e′ velocity (22 cm/s), septal bounce, and a dilated noncollapsing inferior vena cava (17 mm) (Figure 2). Chest computed tomography showed increased pericardial thickness (3.5-4 mm) and focal pericardial calcification (Figure 3). Cardiac magnetic resonance imaging with T1/T2 mapping confirmed pericardial thickening with areas of low signal intensity, consistent with calcification (Figure 4), paradoxical septal motion, and revealed subepicardial late gadolinium enhancement suggestive of post-inflammatory myocardial fibrosis. The latter finding raised the possibility of concomitant myocardial involvement. However, in the absence of clinical or functional evidence of significant myocardial dysfunction, the pericardial process was considered the primary driver of the patient's condition. Cardiac catheterization was also considered for diagnostic workup but was not performed because of sufficient diagnostic data provided by noninvasive tests and no signs of diastolic dysfunction severe enough to proceed with invasive hemodynamic assessment.

**Figure 2:**
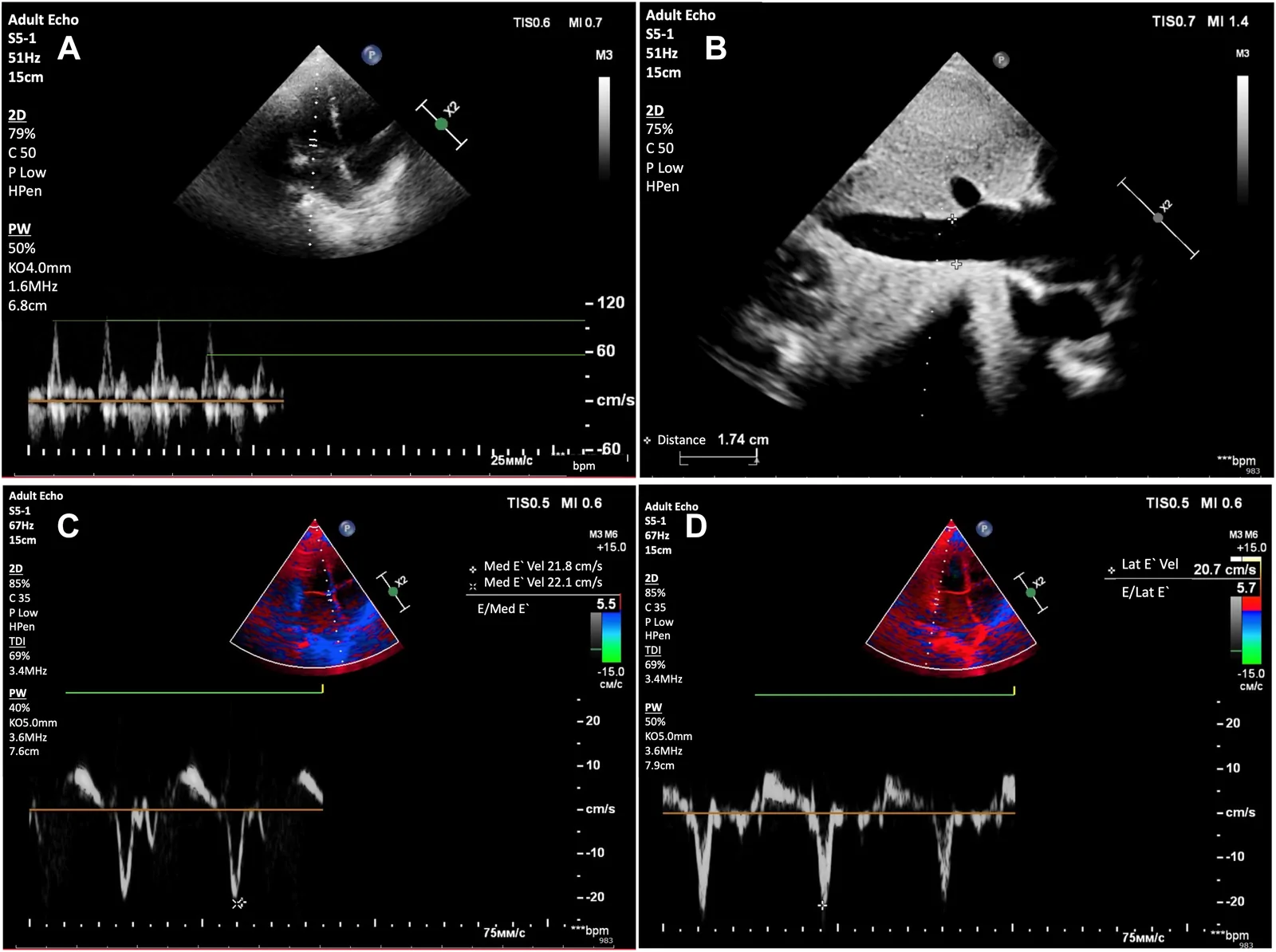
Transthoracic Echocardiography With Dedicated Constriction Assessment Echocardiography demonstrating classical features of constrictive physiology. (A) Pulsed-wave Doppler of mitral inflow shows respirophasic variation >30%. (B) Dilated inferior vena cava (17 mm) with blunted respiratory collapse. (C) Tissue Doppler imaging at the medial mitral annulus reveals increased medial e′ velocity (22 cm/s). (D) Lateral mitral annulus e′ velocity (20.7 cm/s) demonstrates annulus reversus (medial e′ > lateral e′).

**Figure 3:**
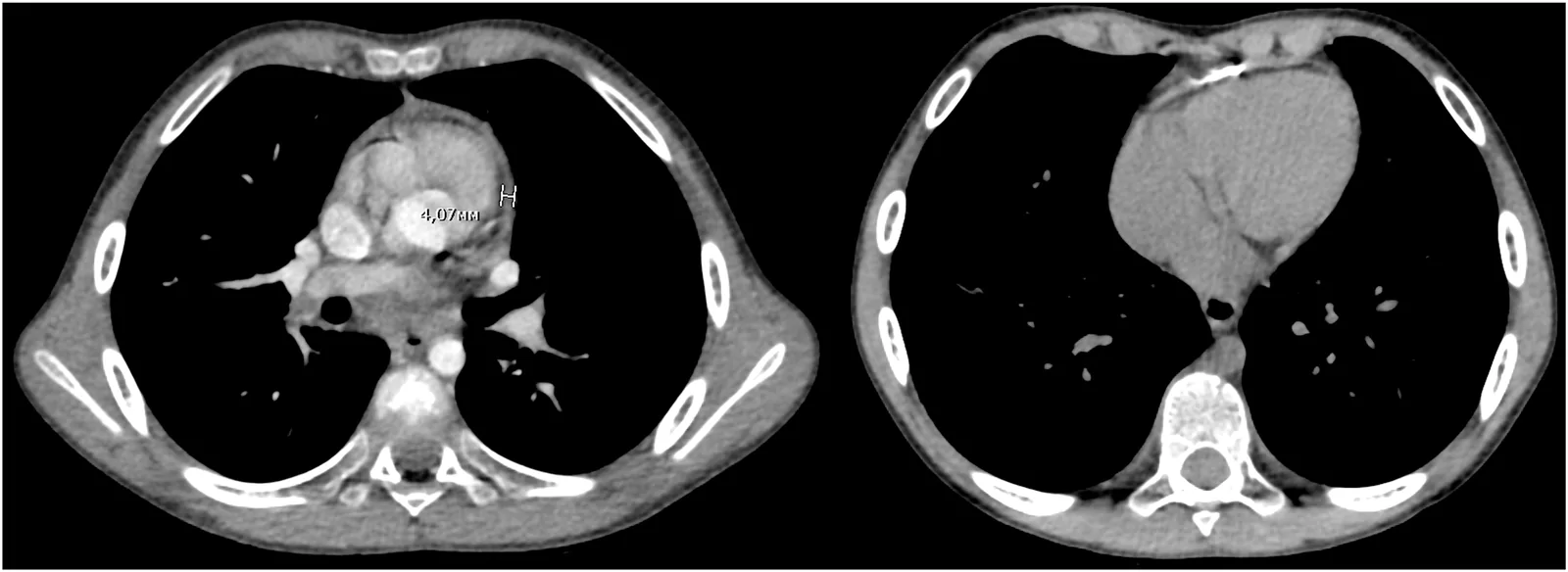
Chest Computed Tomography Demonstrating Pericardial Thickening and Calcifications Chest computed tomography imaging demonstrates diffuse pericardial thickening (3.5-4.0 mm) with focal calcifications adjacent to the right ventricle and the inferoposterior left ventricle. No pericardial effusion is present.

**Figure 4:**
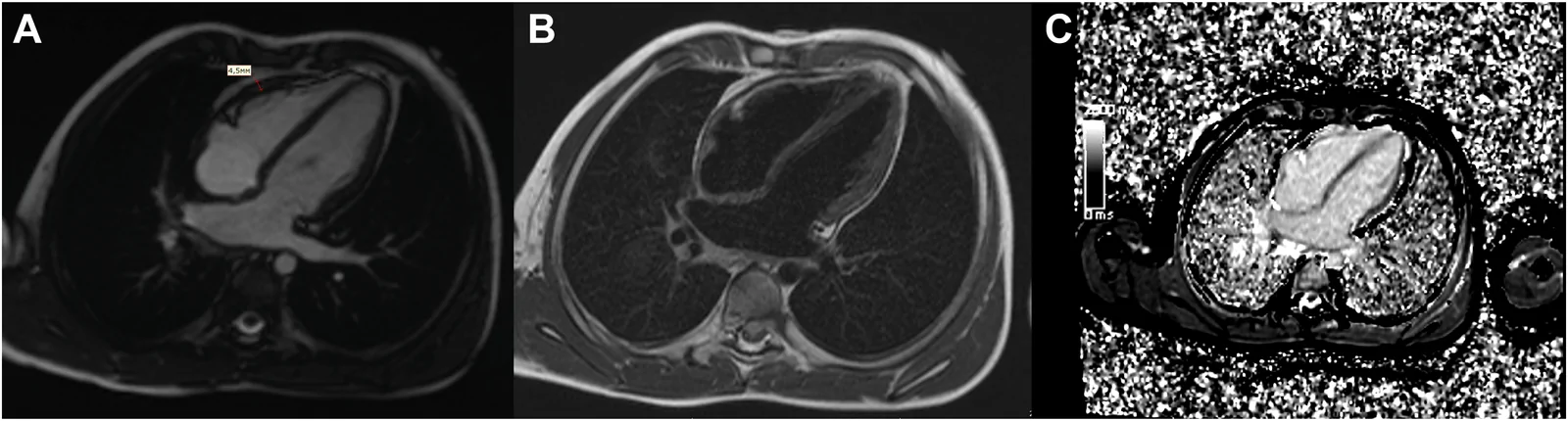
Cardiac Magnetic Resonance With T1/T2 Tissue Mapping (A) Thickened pericardium (4.5 mm) with low-signal areas along the right ventricular free wall, consistent with calcification. (B) T2 weighted imaging shows no evidence of myocardial edema. (C) Native T1 mapping demonstrates mildly elevated T1 relaxation times in most left ventricular segments, with increased extracellular volume in the posterior and inferolateral segments, suggesting diffuse interstitial fibrosis.

## Management (Medical/Interventions)

The diagnosis of chronic constrictive pericarditis (CCP) was established as the underlying cause of liver cirrhosis. Given the absence of active inflammation and the presence of significant organ involvement, the patient underwent pericardiectomy. The surgery was performed via a median sternotomy approach. A calcified area of the pericardium measuring 30 × 7 mm was identified along the inferior aspect of the anterior surface. As no hemodynamic instability or arrhythmias occurred during dissection, pericardiectomy was performed without cardiopulmonary bypass. A markedly thickened, calcified, and myocardium-adherent pericardium was removed (Figure 5). Subtotal pericardiectomy produced immediate hemodynamic improvement, including a reduction in central venous pressure from 18 to 8 to 10 mm Hg intraoperatively. Histopathological examination of the resected pericardium demonstrated areas of fibrosis with focal hemorrhage, lymphohistiocytic infiltration, and calcium deposits, consistent with chronic pericarditis. No specific infectious etiology was identified.

**Figure 5:**
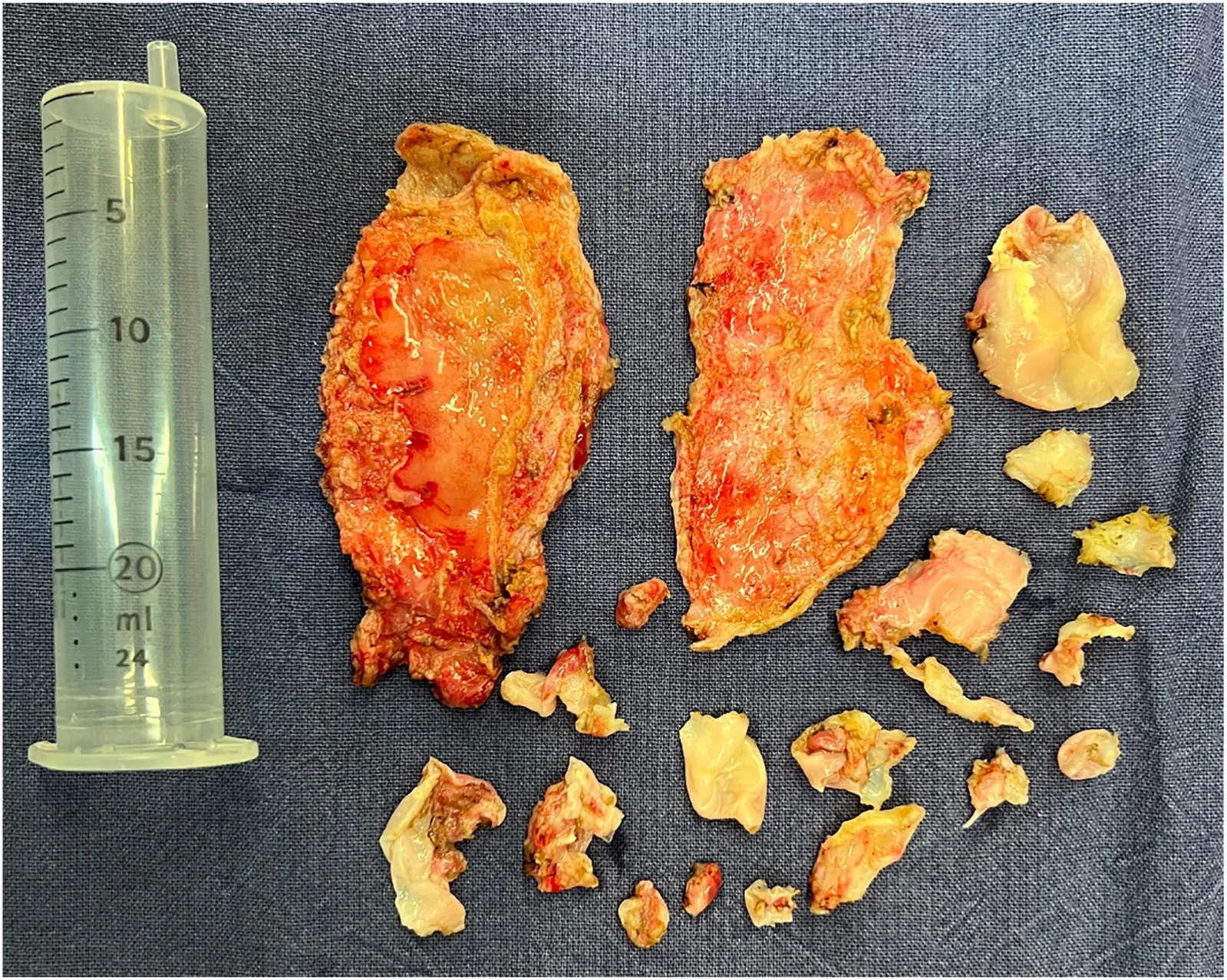
Intraoperative Specimen Resected pericardium is markedly thickened and calcified and was densely adherent to the underlying myocardium.

## Outcome and Follow-Up

The patient had an uneventful postoperative course. Chest tube drainage was 150 mL of transudate on the first postoperative day and 50 mL on the second day, after which drains were removed. The patient was discharged on postoperative day 6. At 3-month follow-up, significant improvement in liver status was observed, with elastography demonstrating METAVIR stage F1 fibrosis. The regression of liver fibrosis after relief of pericardial constriction supports venous congestion as the underlying mechanism of the initial findings consistent with cirrhosis.

## Discussion

CCP is characterized by irreversible pericardial thickening and fibrosis, resulting in significant limitation of ventricular diastolic filling. The etiology in high-income countries is mostly idiopathic, followed by viral and postsurgical causes. The clinical manifestations of CCP are determined by constrictive pathophysiology and include signs of systemic venous congestion, such as jugular venous distention, hepatomegaly, and bilateral lower extremity edema.^2^ Multimodality cardiac imaging, including TTE, cardiac magnetic resonance, and magnetic resonance imaging, is important for diagnosis of pericardial disease and preoperative evaluation. Pericardiectomy remains the definitive treatment of CCP, as supported by both European and American clinical guidelines.^3^^,^^4^

CCP is uncommon in children compared with adults and is therefore rarely considered in the differential diagnosis. In the pediatric population, its presentation may be atypical and dominated by noncardiac symptoms, making diagnosis particularly challenging.^5^^,^^6^ In children, the consequences of late diagnosis are compounded by ongoing somatic development and the risk of irreversible end-organ injury. Clinical presentation of the reported patient was primarily characterized by gastrointestinal manifestations, resulting in the diagnostic delay of nearly 5 years. This case demonstrates the importance of high index of suspicion and awareness of a cardiac etiology in patients with unexplained hepatomegaly and systemic venous congestion, as surgical management can be curative.^7^^,^^8^

Multimodality imaging is essential for diagnosis. However, its interpretation may be affected by the initial clinical framing. In this case, the initial TTE did not identify significant abnormalities, likely due to the absence of clinical suspicion for constrictive physiology. Although key echocardiographic features of constriction, such as respirophasic inflow variation and increased medial mitral e′ velocity, can be detected during standard examination, they require targeted assessment guided by clinical suspicion.^9^

A notable feature of this case is the patient's initial diagnosis with METAVIR stage F4 cirrhosis. The underlying pathophysiology of these findings is related to chronic venous congestion and may be observed in the setting of prolonged right-sided heart failure of any cause. Elevated right-sided filling pressures lead to persistent hepatic venous hypertension, resulting in sinusoidal dilatation, centrilobular congestion, and impaired oxygen delivery to hepatocytes, thereby promoting fibrosis.^10^ However, unlike primary cirrhosis, these changes assessed by elastography do not necessarily reflect irreversible architectural remodeling of the liver. Preserved liver synthetic function, reflected by normal transaminases, albumin, and only mild coagulation abnormalities, was inconsistent with the degree of liver stiffness (40 kPa) and suggested a noncirrhotic mechanism of fibrosis.

The marked regression of liver stiffness from F4 to F1 within 3 months following pericardiectomy is the most striking finding of this case. In primary cirrhosis, stage F4 fibrosis is generally considered irreversible. The rapid improvement observed here is best explained by fibrosis driven by venous hypertension, rather than toxic, viral, or metabolic injury. Following the relief of constriction by pericardiectomy, venous pressures normalized, allowing reversal of congestion-related fibrotic changes. The speed and extent of recovery in this patient may reflect the regenerative capacity of the pediatric liver. Whether similar reversibility can be achieved in adults remains an open question.

## Conclusions

This case highlights that the diagnosis of CCP in pediatric patients can be challenging because of its atypical presentation and low prevalence. However, early recognition is crucial, as timely surgical treatment may lead to significant clinical improvement and reversal of congestion-related liver fibrosis.

**Visual Summary:**
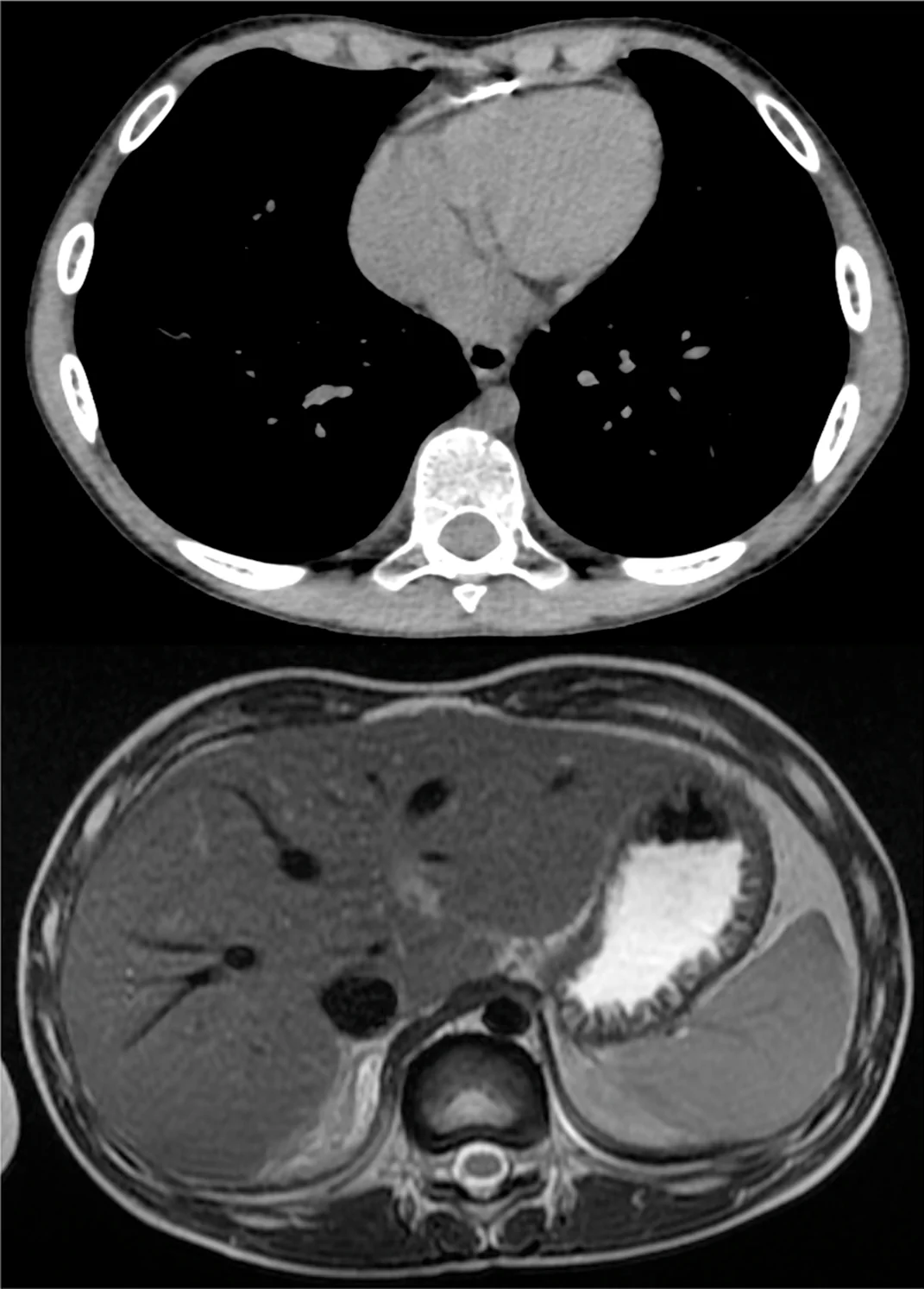
Constrictive Pericarditis Should Be Considered in Children With Unexplained Hepatomegaly and Signs of Systemic Venous Congestion Timely surgical treatment may lead to significant clinical improvement and reversal of congestion-related liver fibrosis.

## Funding Support and Author Disclosures

The authors have reported that they have no relationships relevant to the contents of this paper to disclose.

**Take-Home Messages**

• Constrictive pericarditis should be considered in children with unexplained hepatomegaly and signs of systemic venous congestion.
• Timely surgical treatment may lead to significant clinical improvement and reversal of congestion-related liver fibrosis.
